# Color Duplex Ultrasound Profiling of Native Hemodialysis Arteriovenous Fistula

**DOI:** 10.7759/cureus.91876

**Published:** 2025-09-09

**Authors:** Prashant Sarda, Rohit Sharma, Sovinder Baisoya, Vivek Ruhela, Rajendra Srivastava, Sandeep Kumar, Kiran Kumar Shetty

**Affiliations:** 1 Radiodiagnosis, Shri Guru Ram Rai Institute of Medical and Health Sciences, Dehradun, IND; 2 Radiology, Shri Guru Ram Rai Institute of Medical and Health Sciences, Dehradun, IND; 3 Nephrology, Shri Guru Ram Rai Institute of Medical and Health Sciences, Dehradun, IND; 4 Product Performance Engineering and Post-market Clinical Follow-Up, Meril Life Sciences, Vapi, IND

**Keywords:** arteriovenous fistula (avf), predictor, surgery, ultrasonography (usg), urology

## Abstract

Background

Arteriovenous fistulas (AVFs) remain the preferred vascular access for hemodialysis patients due to their superior long-term patency and lower complication rates. However, AVF dysfunction due to stenosis and thrombosis remains a significant clinical challenge. Color duplex ultrasound (CDUS) has emerged as a valuable, non-invasive tool for AVF assessment, but its accuracy compared to digital subtraction angiography (DSA) remains debated. This study aims to evaluate the diagnostic reliability of CDUS in detecting AVF dysfunction and compare its findings with DSA.

Methods

A prospective observational study was conducted over 18 months at Shri Guru Ram Rai Institute of Medical & Health Sciences, Dehradun, including 57 hemodialysis patients undergoing AVF evaluation. CDUS parameters, including peak systolic velocity (PSV), flow volume (FV), and draining vein diameter, were assessed and compared with DSA findings. Diagnostic performance, inter-method agreement, and key hemodynamic determinants of AVF function were analyzed.

Results

CDUS demonstrated high sensitivity (93.35%) and specificity (92.86%) in detecting stenosis, with 100% agreement with DSA in identifying stenosis location and severity. Functional AVFs had larger draining vein diameters (6±1.1 mm vs. 2.9±0.7 mm, p=0.001) and lower FVs, emphasizing the importance of hemodynamic balance in AVF maturation. Stenotic length significantly impacted FV, with longer stenotic segments (>6 cm) associated with reduced flow (p=0.023).

Conclusion

CDUS is a highly accurate and non-invasive modality for AVF surveillance, demonstrating strong agreement with DSA. Given the absence of standardized Doppler criteria, further research should focus on refining hemodynamic thresholds and integrating AI-assisted ultrasound analysis for improved diagnostic precision.

## Introduction

Arteriovenous fistulas (AVFs) remain the vascular access of choice for patients with end-stage renal disease (ESRD) undergoing hemodialysis due to their superior long-term patency, lower infection rates, and reduced thrombotic complications compared to arteriovenous grafts and central venous catheters [1,2]. Despite these advantages, the clinical success of AVFs is frequently compromised by complications such as primary non-maturation, stenosis, and thrombosis, which can lead to inadequate dialysis, frequent interventions, and increased healthcare costs [3,4]. Early detection and management of these complications are essential to prolong the functional lifespan of the vascular access and improve patient outcomes [5].

Physical examination, while fundamental in the initial assessment of AVF function, is often limited by its subjective nature and inability to detect subclinical abnormalities that precede clinical failure [6]. In recent years, Doppler ultrasound (USG) imaging has emerged as a reliable, non-invasive diagnostic tool, providing real-time vascular hemodynamic information. Quantitative parameters such as peak systolic velocity (PSV), end-diastolic velocity, and flow volume (FV) enable clinicians to detect early hemodynamic alterations predictive of access dysfunction [7]. Furthermore, advancements in high-frequency linear transducer technology have enhanced the resolution and accuracy of these assessments, making it possible to identify subtle changes in vessel wall structure and flow patterns indicative of impending stenosis or thrombosis [8].

Despite the growing role of USG in vascular access surveillance, there remains no universal consensus on specific hemodynamic thresholds that distinguish functioning from non-functioning AVFs [9]. The absence of standardized diagnostic criteria often necessitates a combination of clinical findings and imaging studies to guide therapeutic decisions [10]. Given the significant impact of AVF dysfunction on patient morbidity and healthcare costs, there is an urgent need for robust, evidence-based protocols that can reliably predict access failure at an early stage [11].

This study aims to evaluate the diagnostic accuracy and clinical utility of color duplex ultrasound (CDUS) in assessing native hemodialysis AVFs and compare its findings with digital subtraction angiography (DSA) to determine its reliability in detecting stenosis, thrombotic events, and functional maturation parameters.

AVFs remain the vascular access of choice for patients with ESRD undergoing hemodialysis due to their superior long-term patency, lower infection rates, and reduced thrombotic complications compared to arteriovenous grafts and central venous catheters [1,2]. Despite these advantages, the clinical success of AVFs is frequently compromised by complications such as primary non-maturation, stenosis, and thrombosis, which can lead to inadequate dialysis, frequent interventions, and increased healthcare costs [3,4]. Early detection and management of these complications are essential to prolong the functional lifespan of the vascular access and improve patient outcomes [5].

Physical examination, while fundamental in the initial assessment of AVF function, is often limited by its subjective nature and inability to detect subclinical abnormalities that precede clinical failure [6]. In recent years, USG imaging has emerged as a reliable, non-invasive diagnostic tool, providing real-time vascular hemodynamic information. Quantitative parameters such as PSV, end-diastolic velocity, and FV enable clinicians to detect early hemodynamic alterations predictive of access dysfunction [7]. Furthermore, advancements in high-frequency linear transducer technology have enhanced the resolution and accuracy of these assessments, making it possible to identify subtle changes in vessel wall structure and flow patterns indicative of impending stenosis or thrombosis [8].

Despite the growing role of USG in vascular access surveillance, there remains no universal consensus on specific hemodynamic thresholds that distinguish functioning from non-functioning AVFs [9]. The absence of standardized diagnostic criteria often necessitates a combination of clinical findings and imaging studies to guide therapeutic decisions [10]. Given the significant impact of AVF dysfunction on patient morbidity and healthcare costs, there is an urgent need for robust, evidence-based protocols that can reliably predict access failure at an early stage [11].

This study aims to evaluate the diagnostic accuracy and clinical utility of CDUS in assessing native hemodialysis AVFs and compare its findings with DSA to determine its reliability in detecting stenosis, thrombotic events, and functional maturation parameters.

## Materials and methods

Study design

This research was conducted as a prospective observational study over a period of 18 months at Shri Guru Ram Rai Institute of Medical and Health Sciences, Dehradun. The study aimed to evaluate the role of CDUS in the assessment of native hemodialysis AVFs and its correlation with DSA. Patients with significant stenosis on DSA underwent treatment with MOZEC™ PTA (Meril Life Sciences Pvt Ltd) as per institutional protocol.

Study population

Patients undergoing evaluation for native hemodialysis AVF function using color Doppler sonography, who completed both CDUS and DSA assessments and provided informed written consent, were included in the study. Individuals who declined participation were excluded.

Diagnostic device details

CDUS was performed using high-resolution linear ultrasound probes (5-13 MHz) available in the department, including Samsung Accuvix-XG (Samsung Group, Suwon, South Korea), Philips Affinity 30 (Philips Healthcare, Andover, USA), and Mindray DC 30 (Mindray Medical International Limited, Shenzhen, China). DSA was conducted using the Allengers Life HP 30x30 FPD system with Allengers Safety Standard for User from Radiation Exposure (A.S.S.U.R.E) protocols to ensure patient and operator safety.

Patient preparation

Proper patient preparation and standardized examination protocols were followed to ensure accurate and reliable results. For CDUS, patients were positioned in a supine or semi-reclined posture with external arm rotation, using minimal probe pressure and ample ultrasound gel for optimal imaging. The scanning sequence involved a systematic evaluation of the feeding artery, anastomotic site, and draining vein, with key measurements including vessel diameter, depth, and compressibility. Spectral and color Doppler imaging were utilized to assess blood flow velocity and identify any stenotic changes. For DSA, a 6F/5F radial sheath was inserted under aseptic conditions. The venous outflow tract was visualized using manual injections of non-ionic contrast material, while arterial inflow and anastomotic sites were evaluated with flow interruption techniques.

Measured parameters

The evaluation included both CDUS and DSA parameters. CDUS parameters assessed PSV at the feeding artery, anastomotic site, and draining vein, along with FV, diameter, and depth of the draining vein. Additional factors such as compressibility, presence of early draining veins, stenosis, thrombus, turbulence, and reversal of flow in the radial artery were also examined. DSA parameters focused on the presence and site of stenosis, percentage narrowing, length of the stenotic segment, and the presence of early draining veins or collateralization.

Diagnostic criteria for arteriovenous fistula maturation and dysfunction

The classification of AVFs as functioning or non-functioning was based on the Kidney Disease Outcomes Quality Initiative (KDOQI) guidelines. CDUS-defined maturation criteria included an FV exceeding 600 mL/min, a draining vein diameter greater than 0.6 cm, and a depth of less than 0.6 cm from the skin surface. Clinical maturation criteria were established based on a blood pump speed greater than 300 mL/min during dialysis, successful cannulation in at least 75% of dialysis sessions over four weeks, and a urea reduction ratio exceeding 70%.

Statistical analysis

Data entry and cleaning were performed using Microsoft Excel and SPSS (version 27). Categorical variables were summarized as frequencies and percentages, while continuous variables were expressed as mean ± standard deviation (SD). Normality of continuous data was assessed using the Shapiro-Wilk test. The Chi-square test, or Fisher’s exact test when the expected cell count was <5, was applied to examine associations between categorical variables. Differences in means between two independent groups were analyzed using the independent t-test, and comparisons across more than two groups were performed using one-way ANOVA. A contingency table approach was employed to calculate the diagnostic accuracy of ultrasonography (USG). A p-value <0.05 was considered statistically significant.

Study approval

The study was approved by the Institutional Ethics Committees (IECs) prior to initiation (Approval Number: SGRR/IEC/74/21).

## Results

Demographics

The age distribution pattern revealed that functioning fistulae were most commonly observed in the 51-60 years age group (18 (43.9%)), while non-functioning fistulae were more evenly spread across the 31-60 years range, each group contributing five (31.3%) cases. In terms of gender, males had a higher proportion of functioning fistulae (28 (68.3%)) compared to females (13 (31.7%)), while the non-functioning group showed an equal gender distribution (8 (50.0%)each). The site of fistula creation showed a marked difference between groups; wrist fistulae were predominant among those that functioned well (28 (68.3%)), whereas elbow fistulae were more commonly associated with non-functioning outcomes (10 (62.5%)). A similar trend was noted in the type of fistula: radiocephalic fistulae were more frequently functional (28 (68.3%)), while brachiocephalic fistulae were more often associated with non-functionality (10 (62.5%)) (Table 1).

**Table 1:** Demographics ^*^Significant. BC: brachiocephalic: RC: radiocephalic

Variables	Study group		Test statistics	P-value
	Functioning fistulae (n, 41)	Non-functioning fistula (n, 16)		
Age				
10-20	4 (9.8)	0 (0)	χ²=1.772	P=0.412
21-30	4 (9.8)	1 (6.3)		
31-40	6 (14.6)	5 (31.3)		
41-50	9 (22)	5 (31.3)		
51-60	18 (43.9)	5 (31.3)		
Gender				
Male	28 (68.3)	8 (50)	χ²=0.1983	P=0.160
Female	13 (31.7)	8 (50)		
Site				
Elbow	13 (31.7)	10 (62.5)	χ²=4.5338	P=0.0332^*^
Wrist	28 (68.3)	6 (37.5)		
Type of fistula				
BC	13 (31.7)	10 (62.5)	χ²=4.5338	P=0.0332^*^
RC	28 (68.3)	6 (37.5)		

Doppler profile

On Doppler evaluation, the draining vein diameter was notably larger in patients with functioning fistulae, averaging 6 mm, compared to 2.9 mm in those with non-functioning fistulae. In contrast, the distance of the vein from the skin surface showed minimal variation between the two groups, with both measuring approximately 3.4 mm to 3.5 mm. A distinct pattern was observed in FV: functioning fistulae exhibited lower FVs, averaging around 57.8 mL/min, while non-functioning fistulae showed markedly higher values, nearing 149.7 mL/min. These observations indicate that a wider draining vein may be associated with successful fistula function, while abnormally high FVs could potentially contribute to functional impairment (Table 2).

**Table 2:** Doppler profile ^*^Significant. FV: flow volume; dDV: diameter of the draining vein

Variable	Study group		P-value
	Functioning fistulae (n,41)	Non-functioning fistula (n,16)	
dDV (mm)	6±1.1	2.9±0.7	Test statistic=10.4456; p=<0.001^*^
Diameter of vein from skin (mm)	3.4±1	3.5±1	Test statistic=-0.3392; p=0.7357
FV (mL/min)	57.8±12.8	149.72±7.2	Test statistic=-27.0096; p=<0.001^*^

Doppler profile of the functioning group

Doppler assessment across different age groups revealed no notable variation in arterial or venous parameters. The feeding artery peak systolic velocity (FA-PSV) remained relatively consistent across age ranges, with only slight fluctuations noted. Draining vein peak systolic velocity (DV-PSV) also followed a comparable pattern, with minimal differences observed between age brackets. FV and the distance of the draining vein from the skin (DS) demonstrated no meaningful age-related trends. Similarly, gender-based analysis showed comparable Doppler profiles among males and females, with only minor differences in FA-PSV, DV-PSV, and FV, none of which indicated any substantial association with fistula function. When comparing fistula types, both brachiocephalic and radiocephalic variants displayed similar Doppler characteristics. Parameters such as FA-PSV, FV, draining vein diameter, and depth from the skin were closely matched between the two types. These results suggest that Doppler-based vascular measurements remain broadly uniform across age, gender, and fistula type in this cohort and may not independently predict fistula functionality (Table 3).

**Table 3:** Doppler profile of the functioning group FA-PSV: feeding artery peak systolic velocity; FV: flow volume; DV-PSV: draining vein peak systolic velocity; BC: brachiocephalic; RC: radiocephalic; DS: diameter of the stenosis; dDV: diameter of the draining vein; An. PSV: anastomotic peak systolic velocity; An. FA: anastomotic flow acceleration

Variable	Study group						
	FA-PSV (cm/sec)	An. PSV (cm/sec)	An. FA	DV-PSV (cm/sec)	FV (mL/min)	dDV (mm)	DS (mm)
Age group							
10-20	201.5±17.7	273.8±21.5	1.3±0.1	256.3±47	595±72	6.4±0.9	3.4±1.3
21-30	170±25.7	243.8±19	1.5±0.4	265.3±39	605±114.4	5.8±0.5	2.6±0.4
31-40	199.5±19.1	274.2±40	1.3±0.2	280.3±23.3	576±82.9	6±0.8	3.9±0.7
41-50	198±27	258.8±43	1.3±0.1	281.4±63	630.3±217.9	6.4±1.5	3.6±1.4
51-60	194.4±28.4	277±49.1	1.4±0.2	258±43.7	543.9±78.7	5.8±1	3.1±0.6
Test statistic	0.9623	0.5644	1.7523	1.4111	0.5082	0.1746	0.5929
P-value	0.440	0.690	0.160	0.250	0.730	0.950	0.67
Gender							
Male	190.5±31.1	263.5±45.8	1.4±0.2	260.2±49.2	566.3±123.3	5.9±1	3.5±1.1
Female	194.4±1.5	259.4±37.9	1.3±0.3	266.8±45	605.5±141.4	6.2±1.2	3.2±0.8
Test statistic	-0.446	0.281	1.266	-0.410	-0.905	-0.839	0.879
P-value	0.668	0.780	0.213	0.684	0.371	0.407	0.385
Fistula type							
BC	193.8±27.7	258.8±40.7	1.4±0.2	264.2±63.5	602.8±188.4	6.1±.4	4.1±2.3
RC	190.8±32.7	263.8±44.7	1.4±0.2	261.4±39.3	567.5±91.7	6±0.9	3.1±2.8
Test statistic	0.286	-0.342	0.000	0.147	0.641	0.382	1.122
P-value	0.776	0.734	1.000	0.885	0.531	0.705	0.269

Doppler profile of a non-functioning group

In the non-functioning fistula group, Doppler assessment across various age categories did not reveal any consistent trends or significant variations in hemodynamic parameters. The FA-PSV and DV-PSV exhibited fluctuations across age groups, but without a discernible pattern or clinical relevance. FV values varied modestly with age, ranging from lower averages in older individuals to slightly higher values in younger ones, though without a meaningful difference. Similarly, the diameter of the draining vein (dDV) and its depth from the skin remained comparable across all age categories, indicating no age-related influence on these Doppler parameters. Gender-based analysis also reflected similar findings; both male and female patients demonstrated comparable FA-PSV, DV-PSV, and FV, along with nearly equivalent draining vein diameter and depth from the skin (Table 4).

**Table 4:** Doppler profile of the non-functioning group FA-PSV: feeding artery peak systolic velocity; FV: flow volume; DV-PSV: draining vein peak systolic velocity; DS: diameter of the stenosis; dDV: diameter of the draining vein; An. PSV: anastomotic peak systolic velocity; An. FA: anastomotic flow acceleration

Variable	Study group						
	FA-PSV (cm/sec)	An. PSV (cm/sec)	An. FA	DV-PSV (cm/sec)	FV (mL/min)	dDV (mm)	DS (mm)
Age group							
10-20	292.3±4.2	79.2±12.1	3.6±1.8	37±2.3	161±61.1	3.2±0.6	5.1±1.1
21-30	290±5.1	78±10.3	3.7±1.2	38±6.3	164±58.6	3.0±0.7	4.9±0.89
31-40	286.2±40.3	55±17.76	5.64±2.16	84.44±81.7	158±71.2	2.7±0.8	3.42±1.3
41-50	273.1±57.3	53.4±21.4	4.94±1.71	69.2±27.08	168±91.2	2.7±0.5	3.16±0.53
51-60	255.4±39.7	60.6±25.2	5.24±1.69	53.2±38.9	120±65.3	2.4±0.4	3.56±1.02
Test statistic	0.962	1.752	0.564	1.393	0.508	0.263	1.411
P-value	0.44	0.16	0.690	0.256	0.730	0.9	0.25
Gender							
Male	278.37±26	53.2±19.1	5.66±1.9	62.2±33.5	129±65.6	2.78±0.44	3.6±10
Female	267.62±51.4	62.12±21.8	4.67±1.6	71.3±66.5	170.9±76.2	3±0.86	3.3±1.1
Test statistic	0.528	-0.8705	1.127	-0.346	-1.179	-0.644	-0.684
P-value	0.606	0.399	0.279	0.735	0.258	0.530	0.571

Doppler profile analysis based on fistula type, stenosis length, and draining vein diameter

When comparing fistula types, radiocephalic fistulae demonstrated notably higher FVs than brachiocephalic ones, suggesting better hemodynamic efficiency in the former. Despite this, other Doppler parameters such as PSV and stenotic segment measurements remained largely comparable between the two types. The length of stenosis was also an important determinant of flow, with longer stenotic segments associated with progressively lower FVs. However, other parameters like velocity and stenosis depth did not vary meaningfully with stenosis length. Additionally, draining vein diameter significantly influenced Doppler findings. Fistulae with larger draining veins (3 mm-6 mm) had substantially higher FVs compared to those with smaller veins (<3 mm). These larger veins also showed increased depth from the skin and notable differences in the calculated flow ratio, highlighting the importance of adequate vein caliber for optimal fistula performance (Table 5).

**Table 5:** Doppler profile analysis based on fistula type, stenosis length, and draining vein diameter ^*^Significant. BC: brachiocephalic; RC: radiocephalic; dS: diameter of the stenosis; FV: flow volume; S-PSV: stenotic peak systolic velocity; dDV: diameter of the draining vein

Variable	Doppler profile			
	S-PSV (cm/sec)	dS (cm/sec)	Ratio	FV (mL/min)
Fistula type				
BC	278.1±46.3	52.6±21.7	5.77±1.67	122.4±58.53
RC	264±41.4	66.17±16.03	4.18±1.49	195.8±73.07
Test statistic	0.598	-1.282	1.812	2.044
P-value	0.55	0.20	0.07^*^	0.041^*^
Length of stenosis				
1-3 cm	277±40.8	60.17±18.8	4.87±1.32	142.67±74.1
3-6 cm	269±42.5	55.7±21.5	5.38±2.52	147.7±64.8
>6 cm	274.5±67.1	47.5±21.9	6.05±1.34	98.5±21.9
Test statistic	0.440	0.596	0.472	4.539
P-value	0.65	0.56	0.63	0.023^*^
dDV				
<3 mm	272.8±46.7	41.7±12.87	6.71±1.17	91.43±25.9
3-6 mm	272.7±44.1	70.11±16.04	3.98±1	195.44±62.37
Test statistic	0.113	-2.31	3.291	4.291
P-value	0.91	0.021^*^	0.001^*^	0.001^*^

Agreement between Doppler ultrasound and digital subtraction angiography

Ultrasound and DSA showed excellent concordance in identifying both the site and severity of stenosis. Both modalities consistently identified the same anatomical locations of stenosis, with most cases involving the anastomosis alone, followed by juxta-anastomotic segments involving either the vein or artery, and a smaller number involving early draining veins. Agreement was particularly high for estimating the percentage of stenosis, especially in cases exceeding 50%, which were accurately detected by both methods. While there was slightly less consistency in categorizing stenosis based on length, the overall pattern of detection remained similar across imaging techniques. These findings reinforce the reliability of ultrasound as a non-invasive tool comparable to DSA in evaluating critical characteristics of AVF stenosis (Table 6).

**Table 6:** Agreement between USG and DSA DSA: digital subtraction angiography; USG: Doppler ultrasound

Variable	Non-functioning fistula		Agreement percent	Cohen’s Kappa
	USG	DSA		
Site of stenosis				
Only juxta-anastomosis	8	8	100	1.00
Juxta-anastomosis and vein	3	3		
Juxta-anastomosis and artery	1	1		
Early draining veins	2	2		
Length of stenosis				
≤3 cm	3	6	83.33	0.571
3-6 cm	5	4		
>6 cm	4	2		
Percentage of stenosis				
>50%	12	12	100	1.00
<50%	0	0		

Diagnostic performance of Doppler ultrasound

USG demonstrated excellent diagnostic accuracy in identifying stenosis, showing strong alignment with DSA findings. The high rate of true positive and true negative results underscores its reliability as a primary diagnostic modality. Most cases identified as stenotic on ultrasound were confirmed by DSA, reflecting a strong ability to accurately detect disease. Additionally, the method showed good capacity to exclude stenosis when absent, though with slightly lower certainty. Overall, the diagnostic accuracy of USG Doppler was high, supporting its utility as a non-invasive, efficient, and clinically dependable tool for evaluating AVF integrity (Table 7).

**Table 7:** Diagnostic performance of USG Doppler USG: ultrasonography

Statistical value	Value	95% CI
Sensitivity	93.35%	84.19-99.43
Specificity	92.86%	66.13-99.82
Positive likelihood ratio	13.35	2.02-88.35
Negative likelihood ratio	0.05	0.01-0.20
Disease prevalence	75.44%	62.24-85.87
Positive predictive value	97.62%	86.10-99.63
Negative predicative value	86.67%	62.5-96.21
Accuracy	94.74%	85.38-98.90

## Discussion

The demographic analysis reveals key trends in AVF functionality among the study population. A total of 57 patients were included, with 41 (71.9%) having a functioning fistula and 16 (28.1%) classified as non-functioning. Age distribution did not show a significant impact on AVF functionality (p=0.370), yet patients between 41-50 years had the highest proportion of functioning fistulas (64.3%). Notably, site-specific differences were observed, as wrist AVFs were more likely to be functional (82.4%) compared to elbow AVFs (56.5%), a finding that aligns with previous studies emphasizing the superior maturation potential of RC fistulas [9].

Further, gender was not significantly associated with AVF function (p=0.160), though males had a higher proportion of functional fistulas, supporting prior findings that vascular anatomy and vessel integrity may influence AVF success rates differently in men and women [12].

The Doppler profile findings provide critical insights into hemodynamic parameters distinguishing functional from non-functional AVFs. A significantly larger draining vein diameter (6±1.1 mm vs. 2.9±0.7 mm, p=0.001) was associated with functional AVFs, aligning with the KDOQI criteria, which recommend a minimum draining vein diameter of ≥6 mm for successful maturation [9]. Interestingly, FV was paradoxically lower in functioning fistulas (57.8±12.8 mL/min) compared to non-functioning ones (149.72±72 mL/min, p=0.001). This discrepancy suggests that excessively high flow rates in immature AVFs could contribute to hemodynamic instability or venous hypertension, a concept previously discussed by Lee et al., who highlighted the non-linear relationship between FV and fistula success [13].

When stratifying Doppler parameters by fistula type, no significant differences were noted between RC and BC AVFs. However, the RC group exhibited a slightly higher mean FV and anastomotic PSV, although statistical significance was not reached. These findings are in agreement with prior work by Tessitore et al., who found that RC fistulas generally require a longer maturation period but have superior long-term patency [14].

In non-functioning fistulas, FVs were significantly higher in females compared to males (170.9±76.2 mL/min vs. 129±65.6 mL/min, p=0.38), though not reaching statistical significance. This trend warrants further investigation into the role of gender-based vascular remodeling and endothelial function in AVF maturation, as suggested by Chytilova E et al. [15]. Additionally, stenosis length and draining vein diameter had a significant impact on flow dynamics (Table 5), with AVFs exhibiting longer stenotic segments (>6 cm) showing significantly lower FVs (98.5±21.9 mL/min, p=0.023). These findings echo prior research demonstrating that stenosis beyond 50% significantly compromises AVF function, necessitating early intervention [14].

A major strength of this study is its comparison of USG and DSA findings. The 100% agreement between the two modalities for stenosis location and severity underscores the diagnostic reliability of USG. However, the moderate agreement in detecting short stenotic segments (κ=0.571) suggests that DSA may still be superior for assessing smaller or complex lesions. This corroborates findings from Lok et al. [9], who emphasized that a combination of clinical evaluation, USG, and DSA provides the most comprehensive approach to AVF surveillance.

The diagnostic performance of USG Doppler in this study was exceptional, with a sensitivity of 93.35% and specificity of 92.86%, comparable to previously reported values in the literature [15]. The high positive predictive value (97.62%) reinforces the role of USG as a frontline screening tool for AVF dysfunction, although its moderate negative predictive value (86.67%) suggests that some cases of early stenosis may still be missed without confirmatory DSA.

In conclusion, this study reinforces the role of USG as a highly sensitive and specific diagnostic tool for AVF surveillance. However, given the variability in hemodynamic parameters across different AVF types and patient demographics, there is an urgent need for standardized hemodynamic thresholds to improve diagnostic accuracy. Future research should focus on developing AI-assisted ultrasound interpretation methods and validating long-term predictive models for AVF function, which could further enhance patient outcomes.

Strengths

This prospective study offers one of the few direct comparisons between CDUS and DSA, reinforcing the diagnostic reliability of CDUS in the surveillance of AVFs. By evaluating a comprehensive range of hemodynamic parameters, the study provides detailed insights into AVF function and dysfunction. Its real-time, prospective design minimizes retrospective bias and enhances the clinical applicability of the findings. The results hold significant relevance for routine nephrology and vascular surgery practice, supporting early detection of AVF failure and guiding timely, targeted interventions.

Limitations

Despite its valuable findings, the study has certain limitations. The small sample size of 57 patients restricts the generalizability of the results to broader and more diverse populations. We also acknowledge the lack of a sample size calculation. Being a single-center study, it may not capture variations in AVF outcomes across different institutions, healthcare settings, and patient demographics. Additionally, as USG is inherently operator-dependent, variations in sonographer expertise could influence the accuracy and reproducibility of the diagnostic assessments.

## Conclusions

The present study reinforces the role of CDUS as a highly reliable, non-invasive tool for the assessment of AVF functionality and dysfunction. The findings demonstrate a strong correlation between USG and DSA, highlighting its high sensitivity and specificity in detecting AVF stenosis and maturation parameters. Notably, FV and draining vein diameter emerged as key predictors of AVF function, emphasizing the importance of early surveillance to improve long-term vascular access outcomes. However, the observed discrepancy in FV between functional and non-functional fistulas suggests that hemodynamic parameters should be interpreted with caution, as excessive flow may not always indicate adequate maturation.
